# Extracellular microvesicles/exosomes—magic bullets in horizontal transfer between cells of mitochondria and molecules regulating mitochondria activity

**DOI:** 10.1093/stmcls/sxae086

**Published:** 2025-02-14

**Authors:** Mariusz Z Ratajczak, Kannathasan Thetchinamoorthy, Diana Wierzbicka, Adrian Konopko, Janina Ratajczak, Magdalena Kucia

**Affiliations:** Stem Cell Institute at Brown Cancer Center, University of Louisville, Louisville, KY 40202, United States; Department of Regenerative Medicine, Center for Preclinical Research and Technology, Medical University of Warsaw, Warsaw 02-097, Poland; Department of Regenerative Medicine, Center for Preclinical Research and Technology, Medical University of Warsaw, Warsaw 02-097, Poland; Department of Regenerative Medicine, Center for Preclinical Research and Technology, Medical University of Warsaw, Warsaw 02-097, Poland; Department of Regenerative Medicine, Center for Preclinical Research and Technology, Medical University of Warsaw, Warsaw 02-097, Poland; Stem Cell Institute at Brown Cancer Center, University of Louisville, Louisville, KY 40202, United States; Stem Cell Institute at Brown Cancer Center, University of Louisville, Louisville, KY 40202, United States; Department of Regenerative Medicine, Center for Preclinical Research and Technology, Medical University of Warsaw, Warsaw 02-097, Poland

**Keywords:** extracellular microvesicles, exosomes, transfer of mitochondria, mitophagy, signaling nanotubules

## Abstract

Extracellular microvesicles (ExMVs) were one of the first communication platforms between cells that emerged early in evolution. Evidence indicates that all types of cells secrete these small circular structures surrounded by a lipid membrane that plays an important role in cellular physiology and some pathological processes. ExMVs interact with target cells and may stimulate them by ligands expressed on their surface and/or transfer to the target cells their cargo comprising various RNA species, proteins, bioactive lipids, and signaling nucleotides. These small vesicles can also hijack some organelles from the cells and, in particular, transfer mitochondria, which are currently the focus of scientific interest for their potential application in clinical settings. Different mechanisms exist for transferring mitochondria between cells, including their encapsulation in ExMVs or their uptake in a “naked” form. It has also been demonstrated that mitochondria transfer may involve direct cell-cell connections by signaling nanotubules. In addition, evidence accumulated that ExMVs could be enriched for regulatory molecules, including some miRNA species and proteins that regulate the function of mitochondria in the target cells. Recently, a new beneficial effect of mitochondrial transfer has been reported based on inducing the mitophagy process, removing damaged mitochondria in the recipient cells to improve their energetic state. Based on this novel role of ExMVs in powering the energetic state of target cells, we present a current point of view on this topic and review some selected most recent discoveries and recently published most relevant papers.

## Graphical Abstract

**Graphical Abstract:**
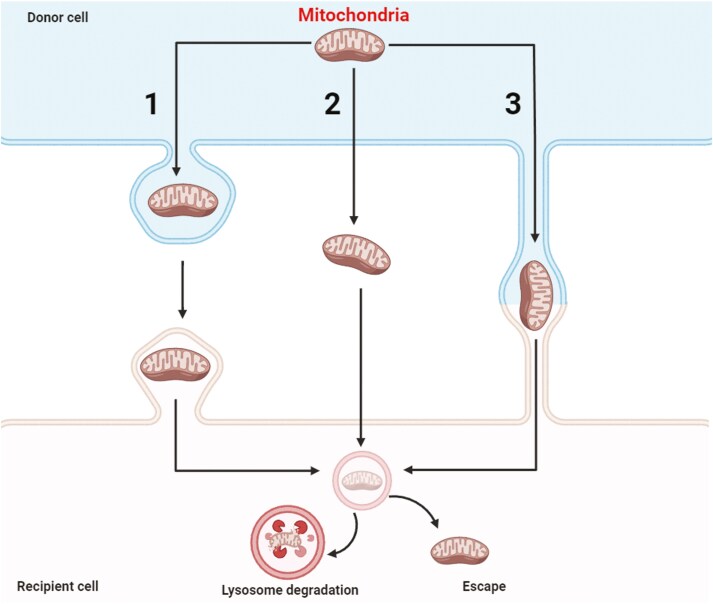

**Significant statement**

It is an invited review of the transfer of mitochondria between cells. It is a hot topic in current biological research

## Introduction

**Figure 1.**
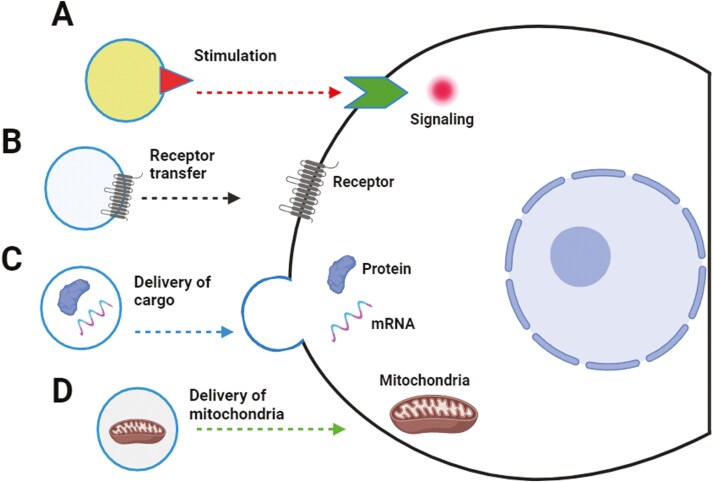
Biological effects of ExMVs. ExMVs may interact with receptors expressed on target cells by surface-expressed ligands (A), transferring receptors to the target cells (B), delivering cargo containing mRNA, miRNA, proteins, or other biomolecules from one cell to another (C), and finally, transferring mitochondria embedded in mito-ExMVs to the target cells (D).

To better understand mechanisms that regulate multicellular organisms’ biology, we must explore cell-cell communication pathways at the single-cell level, including the early developmental role of extracellular microvesicles (ExMVs).^1-4^ This first “communication language” emerged during evolution to exchange biological information between cells. ExMVs are small circular structures surrounded by a lipid membrane that interact with target cells. They may stimulate them directly by ligands encrusted on their surface and/or transfer into these cells’ cargo comprising various RNA species, proteins, bioactive lipids, and signaling nucleotides.^1-4^ ExMVs can also hijack some organelles from the cytoplasm of producing cells, including mitochondria^5-8^ (**Figure 1**). This possibility opens a new area of investigation to understand the role of mitochondria transfer between cells as an important modulator of intracellular energetic performance.

ExMVs have become a focus of significant scientific interest in recent years. They were first discovered by microscope in blood plasma and supernatants from the cell cultures and interpreted as a kind of “cellular dust.”^9^ Understanding their biological involvement in physiological and pathological processes took time.^4^ Twenty years back, we proposed that ExMVs may transfer mRNA and proteins from one cell to another to change their biological function and phenotype.^10^ After an initial wave of skepticism, other investigators have confirmed this fundamental cell-cell communication mechanism.^11-13^ It is the best example of how sometimes novel concepts in science need time to overcome initial disbelief before becoming embraced by the research community. The larger ExMVs are derived from the budding of the cell surface membrane, and smaller ones, called exosomes, are generated for release out of cells from the endosomal compartment.^14-16^

## ExMVs in cell-cell communication and horizontal transfer of bioactive molecules.

ExMVs are composed of an outer lipid bilayer and thus can be considered “physiological liposomes,” in which a surface phospholipid bilayer surrounds the inner content.^1-4^ The cargo of ExMVs is composed of mRNA, miRNA, noncoding RNAs, proteins (eg, enzymes, signaling components, transcription factors), bioactive lipids (eg, sphingosine-1-phosphate, prostaglandins, leukotrienes), signaling nucleotides (eg, extracellular signaling adenosine triphosphate—ATP, adenosine), and some metabolites.^4,17-19^ Due to their unique role in exchanging material and transmitting information between cells, ExMVs emerge as essential modulators of the biological function of targeted cells. Thus, they may be considered as (1) signaling platforms when they stimulate cells with ligands embedded in their outer lipid layer, (2) cell-surface phenotype “modifiers” if they transfer cell membrane receptors to the other cells, and finally (3) cargo-delivery packets that transfer bioactive compounds, genetic material or even some organelles^1-5^ (**Figure 1**). ExMVs are detectable under steady-state conditions in all biological fluids, including blood, lymph, intercellular fluid, cerebrospinal fluid, urine, sperm, bile, synovial fluid, saliva, and breast milk. Based on this, cell-cell communication by small vesicles involves all organs and tissues in the body. In pathologic situations, the number of ExMVs increases, and these pathology-associated ExMVs differ also in molecular composition.

There are two primary mechanisms for release from the cells ExMVs of different sizes. The larger ones, which measure up to 1000 nm in diameter, are derived by the outward budding of the cell surface membrane. In contrast, the smaller ExMVs, up to 150-200 nm in diameter, called exosomes, originate from the endosomal membrane compartment by inward pinching off from the endosomal intraluminal invaginations into vesicles residing in cytosol known as multivesicular bodies (MVB). These vesicles can release their content enriched in small exosomes after fusion with the plasma membrane into the extracellular space.^11-13^ Another source of small exosomes is the Golgi apparatus. This cell organelle helps process and package proteins and lipid molecules, especially proteins destined to be exported from the cell, where these small circular structures are released along with their content from the cells.^4^ Today, on PubMed, there are more than 3000 hits for term extracellular microvesicles and more than 34 000 for term exosomes. This contributes to some confusion in the field as ExMVs and exosomes are released from the cells simultaneously as a part of the widely understood secretome. While it is possible to study the effects of purified populations of large and small extracellular vesicles in experimental settings, their biological effects in vivo are additive due to the cooperative action of microvesicles of different sizes.^4^ Nevertheless, the larger microvesicles seem more critical in transferring cell organelles, including mitochondria.^5^

There are also some differences in the molecular composition of larger ExMVs and smaller exosomes. While ExMVs express CD40, selectins, integrins, cellular receptors, and cytoskeletal proteins, and their membranes are highly enriched in cholesterol, phosphatidylserine, and diacylglycerol, exosomes express specific surface-expressed markers, such as the tetraspanin proteins (eg, CD63/CD9 and CD81), thermal shock proteins (HSP70/90), and major histocompatibility class I antigens.^4,17^ Nevertheless, in this review, we will use the common term ExMVs to describe both large and small vesicles for simplicity.

The current experimental strategies to detect and visualize ExMVs include nanoparticle tracking analysis that calculates size distributions and numbers of ExMVs in suspension, electron microscopy-based approaches to visualize their morphological structure, and flow cytometry combined with antibodies directed against selected surface markers.^4,9^ The more advanced strategies that allow the analysis of molecular signatures of ExMVs are based on the characterization of their cargo (mRNA species, protein, bioactive lipid content, and metabolites) by employing “omics” technologies aimed at the detection of (1) mRNA species (transcriptomics), (2) proteins (proteomics), (3) lipids (lipidomics), and (4) metabolites (metabolomics).^4,9,17,18^

## Mitochondria and their role as the “transferable mini powerplants” between the cells.

Mitochondria are autonomous or semi-autonomous organelles because they contain DNA and synthesize about 10% of their proteins.^15^ Moreover, their DNA is similar to that of the genome of bacteria. In more detail, the human mitochondrial genome is a circular DNA molecule of about 16 thousand base pairs that encode 37 genes. From these 37 genes, 13 are responsible for the subunits of respiratory complexes: I (NADH-dehydrogenase), II (succinate dehydrogenase), III (cytochrome c), IV (cytochrome oxidase), and V (ATP synthase). Other 22 genes encode mitochondrial transfer RNAs, and two encode ribosomal RNA.^16^

**Figure 2.**
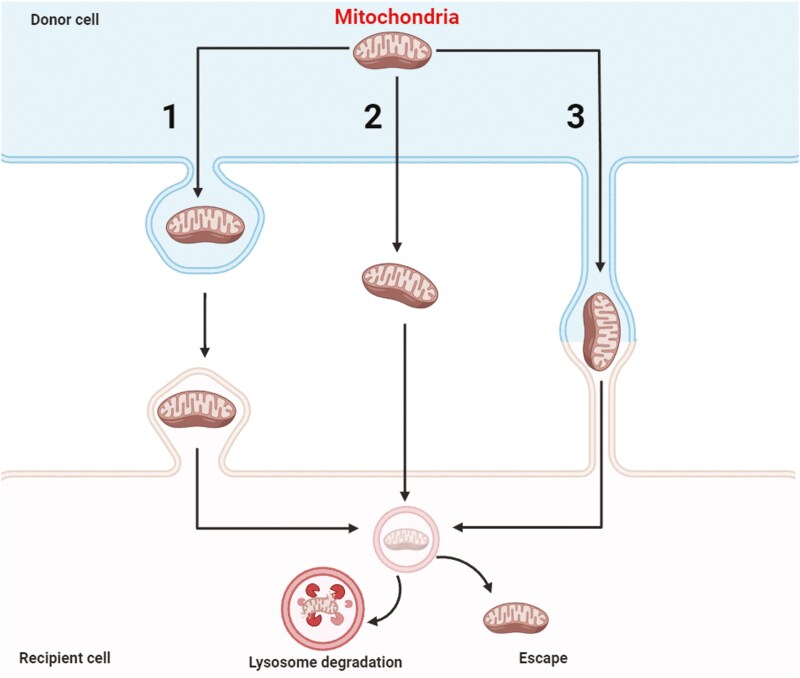
Different ways of horizontal transfer of mitochondria. (1) Mitochondria could be released from producing cells in a RaB7-GDP-regulated manner and internalized and embedded in mito-ExMVs into recipient cells. (2) Mitochondria could also be transferred in the “naked” non-microvesicles associated free from. This uptake could be facilitated after capture by heparan sulfate expressed on recipient cells. (3) Finally, mitochondria could be transferred to the neighboring cells by signaling nanotubules. After transfer into recipient cells, mitochondria could be integrated into the existing mitochondrial network or degraded in lysosomes. In addition to mitochondria, ExMVs may transfer proteins and miRNA into target cells, enhancing the biological function of mitochondria already present in recipient cells.

Overall, mitochondria are envisioned to be evolutionary “travelers.” It has been postulated that these complex organelles involved in energy metabolism, biosynthesis, and stress control are, in fact, proteobacteria that were hijacked by the host eukaryotic cells at the early stages of evolution.^5,15,16,20^ Based on this, the mitochondria in eukaryotes, including animal-, plant-, and fungi cells, similarly to chloroplasts in plants, are most likely evolved from engulfed prokaryotes that lived as independent organisms. At some point in evolution, eukaryotic cells engulfed aerobic prokaryotes, which led to an endosymbiotic relationship, gradually developing into a functional mitochondrion. This may explain the relative easiness of their horizontal transfer between cells. This phenomenon may occur after their (1) encapsulation into extracellular microvesicles (ExMVs) studded with CD9, CD63, CD81, and LC3, and released in a Rab7–GDP-dependent manner for capture by recipient cells, (2) the release as free “naked” mitochondria to be captured by recipient cells in a heparan sulfate (HS)-dependent manner, and (3) finally, they could move inside signaling nanotubules established as communication routes between cells that depend on Cx43, GAP43 and the Rho-GTPase Miro1 shuttle (**Figure 2**).^5^

The mitochondria transfer has also been postulated for smaller ExMVs called exosomes; however, taking into consideration the size of intact mitochondria that are depicted as stiff, elongated cylinders with a diameter of 0.5–1 μm, resembling bacteria, it is clear that the larger ExMVs play in this phenomenon more critical role.^15,16,20^ Up to 2000 mitochondria in cells with active energy consumption, such as cardiomyocytes, represent 30% of cell extract.^8^ Unlike nuclear DNA, which is inherited from both parents, mitochondrial DNA is usually inherited during fertilization from oocytes, where these organelles are abundant. However, both oocyte and sperm cells contain mitochondria with mitochondrial DNA inside; after fertilization, the mitochondria from the sperm, including their DNA, are almost always eliminated.

In this review, we will highlight ExMVs’ novel role in powering the energetic state of target cells. We will discuss the current point of view on this topic and present some seminal recent discoveries. We will also address current trends in employing ExMVs containing mitochondria, “mito-ExMVs,” and “naked” mitochondria isolated from cells or biological fluids in experimental and clinical settings.

## Release of mitochondria from the cells

As mentioned above, mitochondria, as postulated by evolutionists, were incorporated 1.5 billion years ago from an ancient prokaryote into eucaryotic cells by a developmentally ancient endosymbiotic mechanism in which “bacteria” have been captured to facilitate cell metabolism and more efficient energy production.^5,16^ This affirms the critical role of these organelles in cell metabolism by providing energy due to oxidative phosphorylation (OXPHOS) in the form of an energy transfer molecule that is adenosine triphosphate (ATP). Mitochondria are also involved in cell signaling by releasing reactive oxygen species (ROS) that oxidize cysteine and methionine residues in selected transcription factors, histones, enzymes, and structural proteins.^21,22^ This modification of proteins within the “hormetic” beneficial zone^23,24^ may positively regulate cells’ responsiveness to stress and regulate cell signaling, metabolism, proliferation, and immune responses.^21,22^ The number of these important organelles in the cytosol is regulated by fusion and fission mechanisms that ensure maintaining their integrity and proper function.^21^ By fusion, the healthy mitochondria mix their content with damaged ones to rescue their function, and fission helps to remove damaged organelles by mitophagy. Mitochondria are passed on to the daughter cells during cell division (vertical inheritance transfer). However, the so-called horizontal or intercellular transfer of mitochondria exchange between cells occurs and is the main topic of this review.

ExMVs-mediated transfer of functional normal mitochondria has been, for the first time, demonstrated by the team of Dr. Prockop, who used cells that were pretreated with ethidium bromide.^25^ After such treatment, their mtDNA became mutated and depleted, and these mutant cells became incapable of aerobic respiration and growth, except in a permissive medium containing uridine and pyruvate to supplement anaerobic glycolysis. These mutant cells were subsequently co-cultured with normal adult bone marrow-derived nonhematopoietic stem/progenitor cells or skin fibroblasts. The co-cultures between mutant and normal cells produced clones of rescued cells with functional donor cell-derived mitochondria that have been transferred horizontally.^25^

Several reports have been published in this publication’s footstep demonstrating that functional, non-damaged mitochondria can be released from cells in (1) large ExMVs derived by cell membrane budding and (2) small vesicles originating in endosomal compartments involving the formation of multivesicular bodies.^5,16,26-29^ For example, the mechanism of release by cell membrane budding has been demonstrated for thrombin-activated platelets.^29,30^ While large ExMVs play an important role in the transfer of intact mitochondria, the small ones usually contain oxidatively damaged components of these organelles and involve, for example, PINK1-parkin interaction for this to occur, as seen in brown adipocytes.^31,32^ Moreover, ExMVs containing oxidatively damaged mitochondria released by heat-stressed brown adipocytes may be phagocytosed by macrophages for clearance, contributing to metabolic homeostasis. In contrast, osteoblasts release damaged mitochondria in small ExMVs in a process mediated by CD38.^33^ Mito-ExMVs released by these cells facilitate the differentiation of bone progenitors. On the other hand, mito-ExMVs that are secreted by the platelets enhance the pro-angiogenic activity of bone marrow cells.

Similarly, astrocytes-derived mito-ExMVs can release functional mitochondria into hypoxic neurons. This effect is directed by a calcium-dependent mechanism and CD38/cyclic ADP ribose signaling.^34^ In cardiomyocytes, damaged mitochondria are released in larger LC3^+^ vesicles called exophers. This is a new secretory autophagy pathway in which components of LC3 conjugation machinery specify the ExMVs cargo content in the process of LC3-Dependent ExMVs Loading and Secretion (LDELS).^35^ The damaged mitochondria could also be released from cardiomyocytes through smaller ExMVs originating in the endosomal compartment.^16,35^ These examples show that the process of release of damaged mitochondria by ExMVs involves different mechanisms in various cell types.

After delivery to the target cells, the vesicles containing mitochondria can be destroyed in the lysosomal compartment. However, parallel evidence indicates they can escape this mechanism and operate intact in the cytoplasm as a part of the functional intracellular mitochondria network. Mitochondria can also be released from the cells in a “naked form”—non-included in ExMVs and internalized by target cells by micropinocytosis involving cell membrane-expressed heparan sulfate-mediated for a capture mechanism.^36^ Inside the cells, they can bypass a lysosomal compartment, perhaps by using the exact mechanism employed by certain microbes to escape endo-lysosomal capture. Interestingly, it has been recently demonstrated a role of purinergic signaling in the release and uptake of mitochondria embedded in the ExMVs as well as in a “naked” form.^37^ This occurs upon activation of the P2X7 purinergic receptor by extracellular ATP (eATP). This interaction between eATP and P2X7 receptor caused the release of large ExMVs and ExMVs-free mitochondria and promoted their uptake by other cells. Moreover, this transfer increased the energy level in the recipient cells and conferred a pro-inflammatory phenotype.^37^

Evidence for the presence of the “naked” mitochondria secreted from the cells is the lack of ExMVs markers on their surface, such as the expression of mentioned tetraspanins. The competing approach to mito-EXMVs horizontal transfer, transplantation of free mitochondria, has been demonstrated to reduce cardiomyocyte loss in rat models, enhance expression of α-smooth muscle actin and smooth muscle myosin heavy chain II in vascular smooth muscle cells, and significantly increase ATP concentration in lung tissues, leading to improvement of right ventricular contractibility.^20,38^ Moreover, macrophages have been found to modulate inflammatory pain by transferring mitochondria to the sensory neurons.^20,38^ Therefore, it has to be compared and decided which strategies to deliver mitochondria to the cells and tissues are better: (1) by employing mito-ExMVs or (2) by transplantation of “naked” mitochondria.

## Mechanisms of mitochondria transfer between cells

ExMVs during their transfer are internalized by recipient cells, facilitating the transfer of cargoes, including mitochondria, through various mechanisms, including (1) fusion with the recipient cell membrane or (2) internalization due to clathrin-mediated endocytosis or (3) phagocytosis.^1-4,9^ The third possibility for transferring ExMVs and mitochondria between cells is creating signaling nanotubules between communicating cells.^5^ These cytoskeletal protrusions extend from the cell membrane to connect over long distances to other cells. This distance between cells is sometimes over 100 μm, and the diameter of signaling nanotubules ranges from 0.05 μm to 1.5 μm.^5^ However, signaling nanotubules have shown involvement in cell-to-cell communication and transfer of mRNA species, proteins, and mitochondria—similar role-play connexin-43 mediated gap junction channels.^5,16^ We will not discuss these possibilities in more detail since our review focuses mainly on mitochondria transfer involving ExMVs. However, the mechanism of mitochondria transfer by signaling nanotubules has been recently postulated to play an important role in transferring mitochondria from mesenchymal stromal cells to endothelial progenitors, resulting in their better engraftment and function, thus offering a new strategy for vascular regeneration.^8^ However, this intriguing type of crosstalk between cells requires further studies and was recently addressed in the context of mechanisms promoting mitophagy.^39^

## Biological effects of mitochondria transfer by ExMVs (mito-ExMVs)

Intercellular horizontal transfer of mitochondria has been shown to function in energy-demanding tissues and cells, including neurons, cardiomyocytes, renal tubular epithelia, corneal epithelia, and recently in poorly vascularized cartilage tissue.^40^ To support this, recent studies have shown that transferring mito-ExMV and free mitochondria between different cell types, including their transfer from MSCs to lung alveolar epithelial cells and astrocytes to neurons, is crucial for tissue repair. Accordingly, while mitochondrial transfer from bone marrow-derived stromal cells to pulmonary alveoli protects against acute lung injury,^41^ transferring these organelles from astrocytes to neurons after stroke mitigates brain tissue damage.^42^ Transplantation of mitochondria has been reported to positively affect Parkinson’s disease,^43^ where allogeneic/xenogeneic transplantation of peptide-labeled mitochondria restored mitochondria functions and attenuated 6-hydroxydopamine–induced neurotoxicity. Transplantation of mitochondria also showed promising results in mitigating myocardial infarction in pigs^44^ and, more importantly, in pediatric patients.^45^

Different methods are employed to increase the production of ExMVs by parental cells. One is based on genetic manipulation of the ExMV’s biogenesis and release pathways. These genetic engineering techniques require targeting the major genes involved in exosome biogenesis and release. The other method involves pretreating producer cells, changing the culture method, or adding different additives to the medium. Numerous studies have shown that cells secrete more exosomes and enhance the therapeutic potential in hypoxic conditions or after adding specific growth factors and cytokines to the producing cells.^46^ An important role here is the activation of the HIF-1a transcription factor.^46^ It has also been reported that three-dimensional cultures of producing cells have a beneficial role in enhancing the yield of ExMVs.^46^

At the same time, attempts are made to increase mitochondrial load in ExMVs by increasing mitochondrial biogenesis in the donor cells.^46-48^ Exposure of NIH/3T3 fibroblasts or human brain endothelial cells (BEC) using resveratrol to activate peroxisome proliferator-activated receptor-gamma coactivator-1a (PGC-1a) involved in the biogenesis of mitochondria resulted in the incorporation of a more significant number of mitochondria into secreted mito-ExMVs.^46,47^ On the other hand, the selective packaging of mitochondria into ExMVs was promoted by optic atrophy 1 (OPA1) and sorting nexin 9 (Snx9) proteins.^48^ The detailed molecular mechanism mediating this effect, however, requires further investigation.

At the same time, attempts have been made to employ free mitochondria non-associated with ExMVs in the therapy.^20,38^ In particular, recent results with intramyocardial injections of autologous mitochondria isolated from non-ischemic skeletal muscles turned out to be safe and associated with promising signs of recovery of myocardial function.^20,38^ This alternative strategy, however, needs further optimization, for example, effective isolation of intact “naked” mitochondria not protected by ExMVs membranes, the best tissue source of cells producing these organelles, and an optimal way of their administration. However, recent advantages with employing MitoPunch, can simultaneously transfer mitochondria to 10^5^ or more recipient cells, significantly improving the throughput and efficiency compared to existing methods.^49^ One of the challenges to be solved is the storage of isolated free mitochondria.^50^ They can maintain activity for 1-2 hours on ice, and later on their outer membrane is gradually damaged. A similar effect happens after they are stored at −80°C,^51^ and the application of cryoprotectants like DMSO and trehalose, even if they improve to preserve outer membrane integrity, on the other hand, impairs mitochondrial function.^52,53^ The isolation, preservation, and clinical application of free mitochondria are presented elsewhere in some excellent reviews.^5,8,20^

We need also to consider that Mito-ExMVs may also have unwanted effects in some pathologies, including cancer. They also can trigger immune responses. Accordingly, platelet-derived ExMVs enriched for mitochondria may provoke chronic lymphocytic leukemia (CLL) by augmenting oxidative phosphorylation (OXPHOS) and stimulating metabolic reprogramming.^30^ Moreover, this metabolic rewiring of CLL cells enhanced their resistance to some cytostatic, including cytarabine. Similarly, platelet-derived mito-ExMVs enhanced malignant features of breast cancer cells, promoting their migration and invasion.^54^ To support this intriguing observation, these metabolic alterations were exclusively dependent on functional mitochondria, as mito-ExMVs with damaged mitochondria inside did not show an effect.^54^ Interestingly, it has also been hypothesized that the transfer of primary neoplasm-derived mito-ExMVs can contribute to developing donor cell-derived hematologic neoplasm after allogeneic hematopoietic transplantation.^28^ On the contrary, in one of the reports, the transfer of normal epithelial mitochondria into human cancer cells inhibited their proliferation and increased the sensitivity of tumor cells to cytostatics.^55^ Finally, tissue factor (TF) expression on ExMVs may lead to unwanted systemic coagulation.^56-58^ ExMVs may also induce some allergic and immune reactions, creating problems with off-target effects.^59,60^

## Regulation of mitochondrial metabolism and function due to the transfer of regulatory ExMVs-derived miRNA and proteins

Recent evidence indicates that the horizontal transfer of mitochondria by mit-ExMVs and the transfer of ExMVs enriched in regulatory miRNAs may modulate mitochondrial energy metabolism in the target cells. It has been reported that ExMVs-derived miRNAs can translocate after cellular delivery to mitochondria and modulate their activity. For example, mir-149 is involved in SIRT3 activation, increases mitochondrial function, and activates mentioned above, PGC-1ɑ involved in mitochondrial biogenesis.^61,62^ Several miRNAs also directly or indirectly target critical enzymes involved in glycolysis regulation or OXPHOS. While, for example, complex V or ATP synthetase of the respiratory chain is targeted by miR-101 and miR-127, complex IV of the respiratory chain is affected by miR-338 and miR-181c.^63^

Moreover, some miRNAs may affect key enzymes of the citric acid or Krebs Cycle, such as citrate synthetase, for example, miR-152, miR-148a, and miR-19a are involved in this phenomenon.^64^ In addition to miRNAs, other cargo components of ExMVs, comprising several proteins, mRNA, and enzymes, affect mitochondrial function. ExMVs may also contain growth factors and cytokines that upregulate mitochondria-expressed anti-apoptotic proteins (eg, Bcl-2) and, at the same time, downregulate some pro-apoptotic ones (eg, Bax, Bad). They can also reduce the release of pro-apoptotic cytochrome c from mitochondria into cytoplasm, which by inducing apoptosis may minimize the effects of mitochondrial injury.^65^

Based on the fact that ExMVs derived from normal cells may have advantages over synthetic liposomes or nanoparticles and may well protect their inside cargo, including mitochondria, by a membrane bilayer, they could be harnessed in gene therapies. Thus, ExMVs modulating the function of mitochondria in the target cells may serve as a therapeutic approach in mitochondria-related pathologies characterized by defective OXPHOS.^5,16,66,67^ These dysfunctions include mutations in genes encoding elements of the mitochondrial electron transport chain, which utilizes a series of electron transfer reactions to generate cellular ATP through OXPHOS. Therefore, mitochondrial defects could be corrected by transferring normal healthy mitochondria by mito-ExMVs or ExMVs enriched for potential therapeutic molecules.^68^

## Transfer of mitochondria to the damaged cells may induce mitophagy to eliminate defective mitochondria from the target cells

Cardiovascular diseases are one of the significant challenges for regenerative medicine. The most important proposed therapeutic strategies include using stem cells to rejuvenate damaged tissues,^69^ injecting angiogenic growth factors to stimulate local vascularization,^70^ and using ExMVs.^8^ Recent research has highlighted a novel role of mito-ExMVs and free mitochondria transfer in regulating target cells’ mitophagy.

Mitophagy is a critical quality control mechanism inside cells that eliminates damaged mitochondria from the “intracellular mitochondrial network.” This process in mammalian cells occurs in a PTEN-induced putative protein kinase 1 (PINK1) and the E3 ubiquitin ligase Parkin-dependent manner. Both proteins accumulate on damaged mitochondria, promoting their segregation for autophagic degradation in a process that involves Parkin-dependent ubiquitination of damaged mitochondrial proteins.^71^

In a recently published paper, Lin et al^39^ reported the importance of mitochondria transfer in inducing mitophagy in endothelial cells in a model of critical limb ischemia. It has been reported that endothelial cells (ECs) could be transplanted as a therapeutic means in vascular medicine. Still, the successful application of these cells required supporting mesenchymal stromal cells (MSCs).^39^ However, the exact mechanism for the positive effect of MSCs still needs to be discovered. In the paper cited above by Lin et al.^39^ the role of mitochondria transfer from MSCs to ECs has been explained differently. The beneficial effect of mitochondria transfer has been, however, explained differently. There were some open questions in several previously published studies in which mitochondrial transfer has been found helpful in improving energy status in the target cells. Firstly, there was uncertainty about whether mitochondria could survive and remain active after transfer and if a low number of transplanted mitochondria could significantly increase aerobic glycolysis in the recipient cells.

To address these intriguing questions, firstly, the exogenous mitochondria transferred by tunneling nanotubes or internalized after proving into extracellular space did not need to remain functional and be incorporated into the endogenous mitochondria pool. They just triggered the mitophagy process once internalized by clathrin-independent macropinocytosis into the recipient cells to eliminate damaged mitochondria in the recipient cells. This improved the bioenergetics of transplanted cells and their contribution to vascularizing damaged tissues. This process was PINK1-Parkin pathway-dependent, and the transfer of a relatively low number of mitochondria was required to activate mitophagy.^39^ As reported, once mitophagy was triggered, the expression of genes regulating mitochondrial biogenesis (*GFM1, PPARGC1B*, *TMEM59*, and *TEAD4*) was significantly upregulated, and the total mitochondrial content increased, followed by a marked increase in aerobic glycolysis and ATP production.^39^

## Conclusions and problems to be solved

Evidence has accumulated that mitochondria function could be modulated in the target cells by the transfer of these organelles from producing cells by (1) mito-ExMVs and (2) mitochondria in the “naked” form or (3) by employing signaling nanotubules. New strategies have been proposed to enhance mitochondria loading to ExMVs, and they need to be further evaluated for efficacy. On the other hand, the cargo of ExMVs may be enriched for molecules that affect the energetic state of mitochondria in recipient cells. Further research is needed to decipher better the molecular signatures of these regulatory ExMVs (RNA species, enzymatic proteins, bioactive lipids). Recently, a new beneficial effect of mitochondrial transfer has been described based on inducing the mitophagy process in the target cells to remove damaged mitochondria from the intercellular mitochondria network to improve their overall energetic state.

Several problems must be solved before mitoExMVs can be employed successfully in the clinic. It is important to mention that, in particular, small ExMVs (exosomes) have a poor zeta potential, and therefore, they aggregate easily and are unstable.^72,73^ This can result in difficulty delivering exosomes, reaching target sites, and identifying their dosing. Another challenge is standardizing the large-scale production of mito-ExMVs and free mitochondria for effective treatment. Thus, it is necessary to optimize rapid and standardized methods for isolating mito-ExMVs, measuring their exact number, and purifying them efficiently from biological fluids. Considering their therapy application, we must develop isolation protocols corroborating GMP standards. This will require reproducibility of their manufacturing. We still need to identify better signals that promote the release of mito-ExMVs from the cells and strategies to control their enrichment in mito-ExMVs. Considering their therapeutic application in the clinic, we also have to consider the potential “off-target” side effects of such therapy we mentioned above, including, for example, the risk of hypercoagulation or triggering of inflammatory reactions. Moreover, gene, miRNA expression, and isomers of miRNAs have been demonstrated to be variable between genders^74-76^and populations,^76^ suggesting that exosome content and physiological impact could also vary between donors. We are also still looking to identify diseases-specific signatures of mito-ExMVs that could employed in the diagnosis and in tracking response to therapy. Nevertheless, there is no doubt that we will continue to see further exciting progress, particularly with new therapeutic and diagnostic applications of mito-ExMVs. Finally, it has to be solved and compared side by side strategies by independent investigators on how to deliver mitochondria to the cells and tissues.^77^ Is it better to deliver mito-ExMVs or use free mitochondria transplantations? Therefore, let us stay alert to these exciting new developments!

## Data Availability

No new data were generated or analyzed in support of this research.
